# Treatment optimization in low- and middle-income countries

**DOI:** 10.7448/IAS.15.6.18080

**Published:** 2012-11-11

**Authors:** D Cooper

**Affiliations:** University of New South Wales, Sydney, Australia

## Abstract

The unprecedented, successful collaborative international effort to provide universal access to HIV care, including effective antiretroviral therapy (ART), has reached a critical time point. The global economic downturn, changing donor priorities and competing priorities in the health sector threaten the capacity of various agencies to maintain support for the continued scale-up of access toward the UN General Assembly-agreed target of 15 million people with HIV/AIDS receiving ART by 2015. This aspiration has recently received added impetus as we have come to understand that treatment acts as prevention by reducing the infectiousness of treated individuals. It is now necessary to review the elements of the success to date, in order to be able to do more with less. These elements include efforts to optimize delivery of HIV care, including ART, in low- and middle-income countries (LMIC); the emergence of new agents and drug classes which have simplified HIV treatment and made broader successful management more achievable; and changes to commencement protocols. Recent studies have indicated that earlier commencement of HIV therapy is beneficial, leading to changes in the recommended ART initiation threshold in LMIC to <350 CD4 T cells/µL. Studies currently underway are investigating approaches to second-line ART in LMIC. The results from these studies will better inform the rollout of effective second-line therapy. In addition, the financial cost of ART makes optimization of dosing an important consideration in LMIC, in order to maximize effectiveness while limiting costs. ART monitoring is also an important priority in LMIC. Efforts to develop simple and reliable technologies that can provide rapid results in the field are underway. The final priority is operational optimization, to ensure service delivery through initiatives such as exploiting economies of scale and the training and retention of health professionals. Although the challenges in LMIC are substantial and evolving, considerable inroads have been and are being made into optimizing HIV treatment in this area, which is crucial in reducing the global impact of the disease.

